# The incidence of surgical site infection following major lower limb amputation: A systematic review

**DOI:** 10.1111/iwj.14946

**Published:** 2024-07-03

**Authors:** Nina Al‐Saadi, Khalid Al‐Hashimi, Matthew Popplewell, Ismay Fabre, Brenig Llwyd Gwilym, Louise Hitchman, Ian Chetter, David C. Bosanquet, Michael L. Wall

**Affiliations:** ^1^ Black Country Vascular Network Dudley UK; ^2^ Colchester Hospital, North Essex NHS Foundation Trust Colchester UK; ^3^ Institute of Applied Health Research University of Birmingham Birmingham UK; ^4^ South East Wales Vascular Network Cardiff UK; ^5^ Hull York Medical School Hull UK

**Keywords:** amputation, diabetes, lower limb, peripheral arterial disease, surgical site infection

## Abstract

Surgical site infections (SSIs) following major lower limb amputation (MLLA) in vascular patients are a major source of morbidity. The objective of this systematic review was to determine the incidence of SSI following MLLA in vascular patients. This review was prospectively registered with the International Prospective Register of Systematic Reviews (CRD42023460645). Databases were searched without date restriction using a pre‐defined search strategy. The search identified 1427 articles. Four RCTs and 21 observational studies, reporting on 50 370 MLLAs, were included. Overall SSI incidence per MLLA incision was 7.2% (3628/50370). The incidence of SSI in patients undergoing through‐knee amputation (12.9%) and below‐knee amputation (7.5%) was higher than the incidence of SSI in patients undergoing above‐knee amputation, (3.9%), *p* < 0.001. The incidence of SSI in studies focusing on patients with peripheral arterial disease (PAD), diabetes or including patients with both was 8.9%, 6.8% and 7.2%, respectively. SSI is a common complication following MLLA in vascular patients. There is a higher incidence of SSI associated with more distal amputation levels. The reported SSI incidence is similar between patients with underlying PAD and diabetes. Further studies are needed to understand the exact incidence of SSI in vascular patients and the factors which influence this.

## 1 INTRODUCTION

Major lower limb amputation (MLLA) is commonly performed in patients with ischaemia, severe infection or following major trauma.^1^ Most people who undergo MLLA have peripheral arterial disease (PAD), diabetes or both,^2^, ^3^ as well as other risk factors for the development of surgical site infection (SSI) such as tobacco use.^4^

SSIs are associated with increased patient morbidity and mortality.^5^ The Centres for Disease Control and prevention (CDC) classifies SSIs into incisional, which are superficial or deep, and organ/space SSIs.^6^ Developing an SSI post‐MLLA can lead to prolonged hospital admission, additional medical therapy such as prolonged courses of antibiotics, and in more serious cases, surgical drainage, and revision of the amputation stump, including amputation to a higher level.^7^ The National Confidential Enquiry into Patient Outcome and Death (NCEPOD) document ‘Major Lower Limb Amputation: Working Together’ published in 2013 reported that MLLA stump complications were not infrequent, with cellulitis occurring in 16.4% and breakdown in 20.4%,^8^ whether this was secondary to infection, ischaemia or a combination of the two was not reported.

Post‐operative wound infection was identified as a core outcome for vascular patients undergoing MLLA in 2020 by focus groups including patients and healthcare professionals.^9^ The James Lind Alliance research priority setting partnership also identified improving clinical outcomes and healing of the amputation stump (residual limb) as two of the top priorities in vascular patients undergoing MLLA in 2021.^10^ Understanding the incidence of SSI in this population is therefore paramount. Currently, the incidence of SSI in patients who have undergone a MLLA for vascular disease is not well documented. The primary objective of this systematic review is to identify the reported incidence of SSI post‐MLLA for patients with underlying PAD or diabetes overall and within 30 days of surgery. Secondary objectives were to determine the incidence of SSI by aetiology (PAD or diabetes), exact amputation level and study type (randomised controlled trial (RCT) vs. observational study).

## 2 MATERIALS AND METHODS

The protocol for this study outlining the objectives of the review, methods of data collection and analysis was prospectively registered with the International Prospective Register of systematic reviews (PROSPERO; CRD42023460645). This systematic review is reported in accordance with the Preferred Reporting Items for Systematic Review and Meta‐analyses (PRISMA) statement.^11^

### 2.1 Inclusion and exclusion criteria and data sources

RCTs, observational cohort studies and case series with >10 cases which met the inclusion criteria below were included in this review. Editorials, conference abstracts, reviews and case reports were excluded. There was no restriction on publication date.

Inclusion criteria:

1. Patients >18 years old
2. Studies including patients undergoing MLLA (above‐knee amputation (AKA), below‐knee amputation (BKA), through‐knee amputation (TKA) or equivalent)
3. Studies including patients undergoing MLLA related to underlying PAD or diabetes
4. Incidence of SSI reported

Exclusion criteria:

1. MLLAs secondary to trauma or malignancy
2. Foot, ankle or other minor amputations
3. Upper limb amputations
4. SSI incidence not reported
5. No English version of the full text is available

### 2.2 Review process: Data collection process, data items, study risk of bias assessment

The search strategy was developed and tested in collaboration with a clinical librarian. The MEDLINE, EMBASE and CENTRAL databases were searched on 13 February 2024 using the search terms [SSI OR SSIs OR surgical site infection OR surgical wound infection OR wound infection] AND [vascular OR arterial OR diabetes OR diabetic OR gangrene] AND [(amputation AND lower limb) OR MLLA OR MLEA OR above knee amputation OR AKA or below knee amputation OR BKA OR through knee amputation OR TKA OR transfemoral OR transtibial].

Studies identified in the searches were exported onto Microsoft Excel. All duplicate articles were removed before screening. Each study was screened according to title and abstract by two reviewers (NAS and KAH), with conflicts resolved by a third reviewer (MLW). Full‐text screening was conducted by two reviewers (NAS and KAH); again, conflicts were resolved by a third reviewer (MLW). For the studies where the incidence of SSI following MLLA related to diabetes and/or PAD could not be extracted (*n* = 7), the authors were contacted and a request for the data was made.

The primary outcome was the reported incidence of SSI post‐MLLA overall and within 30 days. Secondary outcomes were the incidence of SSI based on:

1. The specific level of major amputation reported in the studies (above‐knee amputation (AKA), below‐knee amputation (BKA) or through‐knee amputation (TKA))
2. Study design (RCT/observational)
3. Study arms (intervention/control)
4. Underlying pathology (peripheral arterial disease and/or diabetes)
5. Reported definition used to diagnose SSI (CDC/ASEPSIS/author‐defined)
6. Whether SSI was a primary outcome (including a coprimary outcome or component of a composite outcome)

Data were organised and extracted by two reviewers independently (NAS and KAH), and discrepancies were checked by a third reviewer who was the senior author (MLW). The data collection tool was piloted and refined to ensure the capture of all relevant data. Study risk of bias assessment was undertaken by two reviewers (NAS and KAH). Cochrane's risk of bias tool^12^ was used for RCTs, and the Newcastle‐Ottawa scale^13^ was used for observational studies. All studies were included in the analysis. The SSI incidence has been reported per MLLA for all patients who met the inclusion criteria. For RCTs, the incidence of SSI in both the intervention and control groups was included.

### 2.3 Synthesis methods

The data collection methodology was specifically designed to facilitate the calculation of the actual incidence of SSI per MLLA incision. This was carried out for the studies overall and for the subgroups. Demographic variables, including the percentage of male patients and mean age, were aggregated, where possible, using the weighted mean. Categorical data were compared using the Chi‐squared test. Analyses were performed using Excel (Microsoft) and Prism 10 software version 10.1.0, GraphPad Software LLC.

## 3 RESULTS

**FIGURE 1:**
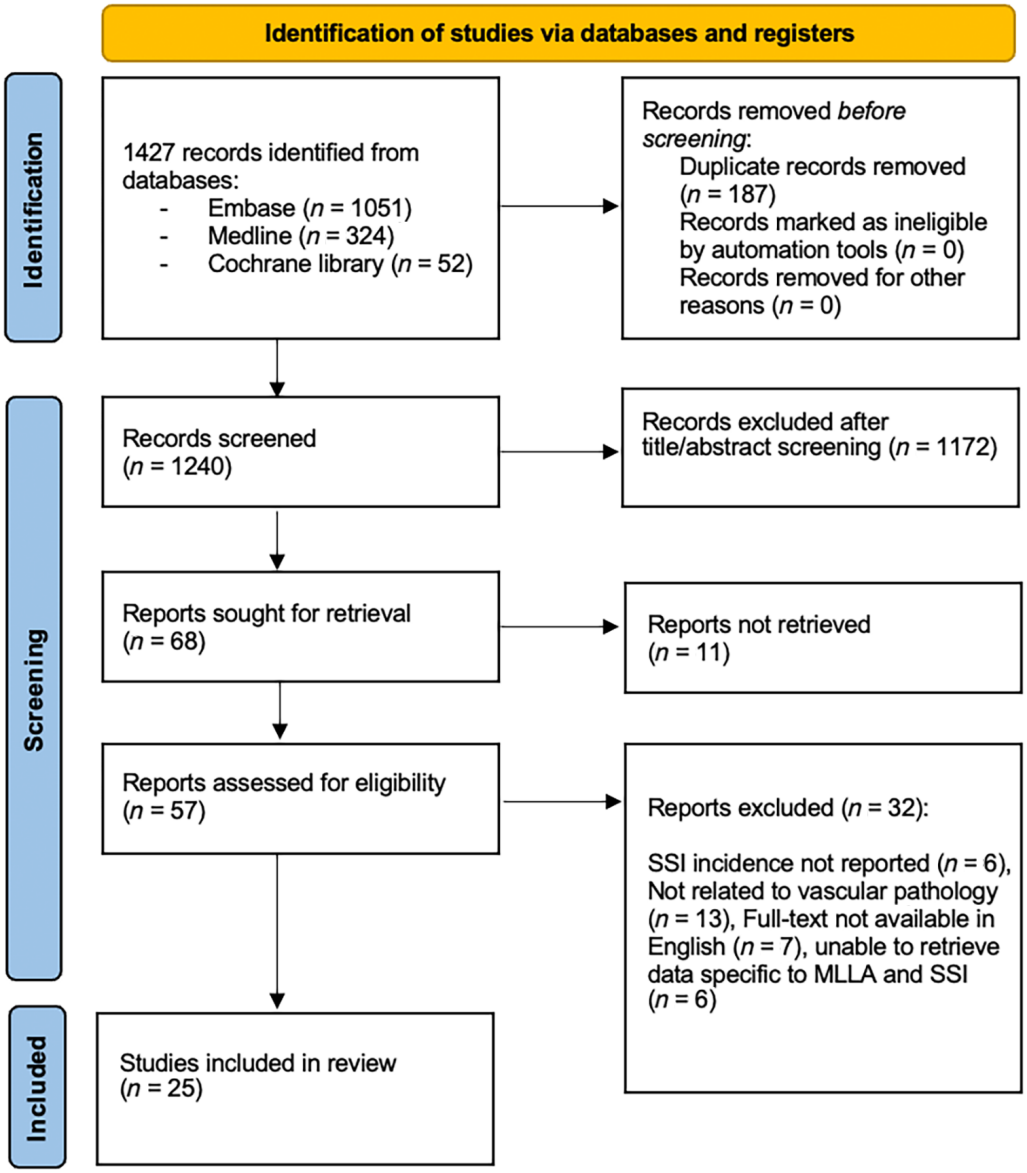
PRISMA flow diagram of literature search of randomised controlled trials and observational studies reporting incidence of SSI in patients who had major lower limb amputations performed secondary to peripheral arterial disease and/or diabetic foot infection. MLLA, major lower limb amputation; PAD, peripheral arterial disease; SSI, surgical site infection.

The initial search performed on 13 February produced 1427 results. After the removal of duplicates, 1240 studies were screened and assessed for eligibility. Following the title and abstract screening, 57 articles underwent full‐text review. After further exclusions, 25 studies were retained for data extraction (Figure 1). Table 1 reports the study demographics.

**TABLE 1:** Study demographic data.

No.	Author	Year	Study date	Location	Study design	Retrospective/Prospective	Centres	No. of patientstotal	No. of patients undergoing MLLA secondary to PAD/diabetes	No. MLLA secondary to PAD/diabetes	Average age (mean/median)	% Male	Follow‐up (mean, days)	Underlying pathology in the study population	Level of amputation in the study
1	Azarbal^14^	2015	2011–2013	Portland, US	Observational	Retrospective	Single	83	83	96	67.3	NR	NR	Both	AKA & BKA
2	Chahrour^15^	2021	2005–2017	Beirut, Lebanon	Observational	Retrospective	Multiple	21 449	21 449	21 449	NR	64.3	NR	Diabetes	AKA & BKA
3	Chang^16^	2021	2018–2019	NY, US	Observational	Retrospective	Single	54	54	54	70	57.4	30	PAD	AKA & BKA
4	Cull^17^	2001	1996–1999	Greenville, US	Observational	Retrospective	Single	12	10	10	63	NR	760	PAD	TKA
5	Dunkel^18^	2012	1995–2010	Geneva, Switzerland	Observational	Retrospective	Single	270	NR	254	NR	NR	60	Both	AKA, BKA & TKA
6	Fearon^19^	1985	1980–1982	Boston, US	Observational	Retrospective	Single	98	98	100	65	57.1	24	Both	BKA
7	Fisher^20^	1988	1984–1886	Texas, US	RCT; one vs two stage amputation	Prospective	Single	47	11	25	NR	NR	60	Diabetes	AKA & BKA
8	Gabel^21^	2017	2013–2015	California, US	Observational	Retrospective	Multiple	2939	2939	2939	66	64	365	Both	AKA & BKA
9	Gwylim^22^	2022	2020–2021	International	Observational	Prospective	Multiple	537	537	537	68	80.4	365	Both	AKA, BKA & TKA
10	Hasanadka^23^	2011	2005–2008	Illinois, US	Observational	Retrospective	Multiple	4250	4250	4250	NR	59.3	30	PAD	AKA & BKA
11	Khouqeer^24^	2020	2009–2018	Texas, US	Observational	Retrospective	Single	182	182	182	65	98	90	PAD	BKA
12	Lim^25^	2018	2005–2012	Illinois, US	Observational	Retrospective	Multiple	7720	7720	7720	70.5	55	30	Both	AKA & TKA
13	Marelli^26^	1994	1985–1990	Montreal, Canada	Observational	Retrospective	Single	111	111	111	71	66.7	365	PAD	BKA
14	Niskakangas^27^	2017	1996–2010	Oulu, Finland	Observational	Retrospective	Single	417	417	534	76	57	365	PAD	AKA & BKA
15	O'Banion^28^	2022	2016–2020	California, US	Observational	Retro & prospective	Single	141	141	141	57	73	351	PAD	AKA & BKA
16	Payne^29^	1989	1986	New South Wales	RCT; pre‐op skin preparation vs no pre‐op skin preparation	Prospective	Single	73	73	80	74	NR	NR	PAD	AKA & BKA
17	Ploeg^30^	2005	1996–2002	Hague, Netherlands	Observational	Retrospective	Single	97	97	122	73	50.5	781	PAD	AKA & BKA
18	Souroullas^31^	2022	2013–2015	Hull, UK	RCT; 5d antibiotics vs 24 h antibiotics	Prospective	Single	152	111	111	66.8	71	30	Both	AKA, BKA & TKA
19	Squire^32^	1982	1973–1977	Boston, US	Observational	Retrospective	Single	75	75	84	NR	100	NR	PAD	AKA
20	Takahashi^33^	2023	2021–2022	Nagoya, Japan	Observational	Retrospective	Single	32	32	32	73	75	30	Both	AKA & BKA
21	Vaddavalli^34^	2023	2021	Chandigarh, India	RCT; CiNPWT vs standard dressing	Prospective	Single	50	50	50	55	74	56	PAD	AKA & BKA
22	Van Niekerk^35^	2001	1994–1996	Dundee, UK	Observational	Prospective	Single	219	219	234	70	60.7	21	PAD	AKA & BKA
23	Vogel^36^	2018	2008–2014	Missouri, US	Observational	Retrospective	Single	2480	2480	2480	68.4	61.3	30	PAD	AKA & BKA
24	Wu^37^	2017	2008–2015	Singapore	Observational	Retrospective	Single	210	210	210	66	64	30	PAD	BKA
25	Yamada^38^	2016	2007–2012	Tokyo, Japan	Observational	Retrospective	Single	8565	8565	8565	73.16	63.5	30	Both	AKA & BKA

### 3.1 Demographic details

The included studies were published between 1982 and 2023. Studies included were RCTs (*n* = 4) and observational (*n* = 21). Overall, across the studies, there were 50 370 MLLAs related to underlying PAD and/or diabetes undertaken in 50 263 patients. The weighted mean age was 70.5 years. The mean percentage of male patients in the studies was 67.6%.

Studies were grouped according to the underlying pathology described in the study's patient cohort: diabetes (*n* = 2), PAD (*n* = 14) or both (diabetes and PAD) (*n* = 9). Studies were also grouped according to the level of major amputation in the study cohort: AKA only (*n* = 1), BKA only (*n* = 4), TKA only (*n* = 4) or AKA ± BKA ± TKA (*n* = 19). When all the studies were pooled, there were 25 015 AKAs, 25 032 BKAs and 323 TKAs across all the studies.

SSI was a primary outcome in 19 studies. The incidence of SSI within 30 days was reported in 12 studies.

Definitions used to report SSI included the CDC criteria (*n* = 7), ASEPSIS score (*n* = 1) and author definition (*n* = 4). In the remaining studies (*n* = 13), it was not reported how SSI was defined.

The severity of SSI (superficial/deep) was reported in 6 studies. Of these studies, 3 used the CDC criteria, 2 did not report how SSI was defined and 1 used an author definition. Table 2 reports study demographics according to outcomes and SSI definition.

**TABLE 2:** Study demographics based on SSI outcomes.

Study	SSI definition	Study's primary outcome	If SSI was a primary outcome, was an intervention assessed?	SSI reported within 30 days (according to SSI definition used)	Was SSI a primary outcome?^a^	Degree of SSI (superficial/deep) reported
1	Author defined (+ve culture or evidence of pus from wound)	Wound Occurrence	No	NR	Yes	No
2	CDC	Surgical site infection	No	Yes	Yes	Yes
3	CDC	Wound complications	Yes—negative pressure versus standard dressing	Yes	Yes	Yes
4	NR	Wound complications and functional outcomes	No	NR	Yes	No
5	Author defined (presence of pus or skin infection requiring ABx or drainage)	Wound infection and dehiscence	No	NR	Yes	No
6	NR	Wound infection and related risk factors	No	NR	Yes	Yes
7	NR	Wound complications	Yes—one versus two staged amputations	NR	Yes	No
8	Author defined (VQI registry—infection with +ve culture or requiring ABx)	Early post‐operative outcomes (including infection)	No	Yes	Yes	Yes
9	CDC	Evaluate the accuracy of predicting short term post‐operative outcomes	No	Yes	No	No
10	CDC	Wound occurrence	No	Yes	Yes	Yes
11	CDC	Wound complications	Yes—the use of an SSI prevention bundle	Yes	Yes	No
12	CDC	Post‐operative outcomes (including infection)	No	Yes	Yes	No
13	NR	Surgical revision and mobility post‐operatively	No	NR	No	No
14	NR	Effects of type of anaesthetic on post‐operative analgesia	No	NR	No	No
15	NR	Post‐operative length of stay, time to receipt of prosthesis and ambulation	N/A	NR	No	No
16	NR	Wound infection	Yes—pre‐operative skin preparation	NR	Yes	No
17	NR	Post‐operative complications	No	NR	Yes	No
18	ASEPSIS	Surgical site infection	Yes—antibiotic therapy 24 hr vs. 5 day and skin preparation	Yes	Yes	No
19	NR	Wound complications	No	NR	Yes	No
20	NR	Wound complications	Yes—negative wound pressure dressings versus standard dressings	Yes	Yes	Yes
21	CDC	Wound complications	Yes—negative pressure dressing versus standard dressings	Yes	Yes	No
22	Author defined (pus discharge with +ve wound culture)	Residual limb complications	No	Yes	Yes	No
23	NR	Readmission within 30 days	No	Yes	No	No
24	NR	Surgical revision	No	No	No	No
25	NR	Post‐operative outcomes (including infection)	No	No	Yes	No

^a^ Primary, coprimary or composite of a primary outcome.

### 3.2 Risk of bias in studies

The reviewers determined one of the RCTs had a high risk of bias, one had some concern for bias and two had a low risk of bias (Table 3). The median Newcastle‐Ottawa score for observational studies was 6, and the range was 3 to 8 (Table 4).

**TABLE 3:** Cochrane risk of bias assessment of randomised controlled trials.

Author	Year	Randomisation process	Deviation from intended interventions	Missing outcome data	Measurement of the outcome	Selection of the reported result	Overall
Fisher	1988	Some concerns	Low concerns	Low concerns	Some concerns	High concerns	Some concerns
Payne	1989	Low concerns	High concerns	Low concerns	Low concerns	Some concerns	High concerns
Vadavalli	2023	Low concerns	Low concerns	Low concerns	Some concerns	Low concerns	Low concerns
Souroullas	2022	Low concerns	Low concerns	Low concerns	Some concerns	Low concerns	Low concerns

**TABLE 4:** Newcastle‐Ottawa score of observational studies.

Author	Year	Selection				Comparability	Outcome			Total	AHRQ outcome
		Representatives of the exposed cohort	Selection of the non‐exposed cohort	Ascertainment of exposure	Demonstration that outcome of interest was not present at start of study	Comparability of cohorts on the basis of the design or analysis	Assessment of outcome	Was follow‐up long enough for outcomes to occur	Adequacy of follow‐up of cohorts		
Azarbal	2015	1	1	1	1	0	1	0	1	6	Poor quality
Chahrour	2021	1	1	1	1	1	1	0	1	7	Good quality
Chang	2021	0	0	0	0	0	1	1	1	3	Poor quality
Dunkel	2012	0	1	0	0	1	1	1	0	4	Poor quality
Cull	2001	0	0	1	0	0	0	1	1	3	Poor quality
Fearon	1985	1	1	1	0	1	0	1	1	6	Good quality
Gabel	2017	1	1	1	0	1	1	1	1	7	Good quality
Gwilym	2022	1	1	1	0	1	1	1	1	7	Good quality
Hasanadka	2011	1	1	1	0	1	1	1	1	7	Good quality
Khouqeer	2020	1	1	1	0	0	1	1	1	6	Poor quality
Lim	2018	1	1	1	0	2	1	1	1	8	Good quality
Marelli	1984	1	1	1	0	0	0	1	0	4	Poor quality
Niskakangas	2017	1	1	1	1	0	0	1	0	5	Poor quality
O'Banion	2022	0	1	1	0	2	1	1	1	7	Good quality
Pleog	2005	1	1	1	0	0	1	1	1	6	Poor quality
Squire	1982	1	1	1	0	0	1	0	0	5	Poor quality
Takahashi	2023	1	1	1	1	1	1	1	0	7	Good quality
Van Niekerk	2001	1	1	1	0	1	1	1	1	7	Good quality
Voegl	2018	1	1	1	0	1	0	1	0	5	Poor quality
Wu	2017	1	1	0	0	0	1	1	1	5	Poor quality
Yamada	2016	1	1	1	0	1	1	1	1	7	Good quality

### 3.3 Overall SSI rates

**FIGURE 2:**
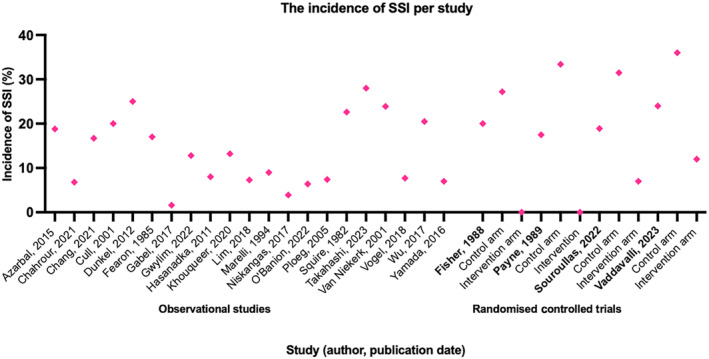
Graph illustrating the incidence of SSI (%) in each of the studies, including the intervention and control arms of the RCTs.

There were 3628 SSIs in 50 370 MLLAs, which equates to an SSI incidence 7.2% per MLLA. This is the incidence of SSI across all the studies during their follow‐up period (21–781 days). The weighted SSI incidence was also 7.2%. Figure 2 represents the incidence of SSI per MLLA in each study.

In studies which reported the degree of SSI, there were more superficial SSIs reported across the studies (1096/28792, 3.8%) compared with deep SSIs (646/28792, 2.2%). In studies which reported the incidence of SSI according to the level of amputation (*n* = 18), the SSI incidence in patients undergoing TKA and BKA was higher than the incidence of SSI in patients undergoing AKA (12.9%, 7.5% and 3.9%, respectively, *p* < 0.001).

In studies where SSI was a primary, coprimary or component of a composite primary outcome, the incidence of SSIs was 7.1% (3285/46378); in studies where SSI was a secondary outcome, the incidence of SSI was 8.5% (343/4013). In studies which used a formal definition to report SSI (*n* = 8), the incidence of SSI was 7.3% (2497/34332). Table 5 summarises the incidence of SSI according to the study type, outcomes and patient characteristics.

**TABLE 5:** The incidence of SSI according to study design, patient cohort and group.

Variable	Observational study and RCT, *n* (%)	Observational studies, n (%)	RCT both arms, *n* (%)	RCT control arm, *n* (%)	RCT intervention arm, *n* (%)
All studies (*n* = 25)					
SSI	3628/50 370 (7.2)	3578/50 104 (7.1)	50/266 (18.8)	43/266 (16.2)	7/266 (2.6)
Superficial SSI	1096/28 792 (3.8)	1096/28 792 (3.8)			
Deep SSI	646/28 792 (2.2)	646/28 792 (2.2)			
Studies that include patients who underwent MLLA related to peripheral arterial disease only (*n* = 14)					
SSI	759/8542 (8.9)	733/8412 (8.7)	26/130 (20)	23/130 (17.7)	3/130 (2.3)
Superficial SSI	219/4314 (17.6)	219/4314 (5.1)			
Deep SSI	130/4314 (3.0)	162/4314 (3.8)			
Studies that include patients who underwent MLLA related to underlying diabetes only (*n* = 2)					
SSI	1462/21 474 (6.8)	1459/21 449 (6.8)	3/25 (12)	3/25 (12)	0/25 (0)
Superficial SSI	841/21 449 (3.9)	841/21 449 (3.9)			
Deep SSI	484/21 449 (2.3)	484/21 449 (2.3)			
Studies that include patients who underwent MLLA related to underlying peripheral arterial disease and/or diabetes (*n* = 9)					
SSI	1407/20 354 (7.2)	1386/20 243 (6.8)	21/111 (18.9)	17/111 (15.3)	4/111 (3.6)
Superficial SSI	36/3071 (11.7)	36/3071 (11.7)			
Deep SSI	36/3071 (11.7)	36/3071 (11.7)			
Studies that include patients who underwent an AKA only (*n* = 1)					
SSI	19/84 (22.6)	19/84 (22.6)			
Superficial SSI					
Deep SSI					
Studies that include patients who underwent a BKA only (*n* = 4)					
SSI	94/603 (15.6)	94/603 (15.6)			
Superficial SSI					
Deep SSI					
Studies that include patients who underwent a TKA only (*n* = 1)					
SSI	2/10 (20)	2/10 (20)			
Superficial SSI					
Deep SSI					
Studies that include patients who underwent an AKA ± BKA ± TKA (*n* = 19)					
SSI	3513/49 673 (7.1)	3463/49 407 (7.0)	50/266 (18.8)	43/266 (16.2)	7/266 (2.6)
Superficial SSI	1087/30 209 (3.6)	1087/30 209 (3.6)			
Deep SSI	641/30 209 (2.1)	641/30 209 (2.1)			
Studies where SSI was a primary outcome (*n* = 19)					
SSI	3285/46357 (7.1)	3235/46 901 (6.9)	50/266 (18.8)	43/266 (16.2)	7/266 (2.6)
Superficial SSI	1091/28 792 (3.8)	1091/28 792 (3.8)			
Deep SSI	646/28 792 (2.2)	646/28 792 (2.2)			
Studies where SSI was not a primary outcome (*n* = 6)					
SSI	343/4013 (8.5)	343/4013 (8.5)			
Superficial SSI					
Deep SSI					
Studies that used incidence of SSI reported within 30 days (*n* = 12)					
SSI	2799/40 038 (7.0)	2766/39 877 (6.9)	33/161 (20.5)	26/161 (16.1)	7/161 (4.3)
Superficial SSI	1092/28 724 (3.8)	1092/28 724 (3.8)			
Deep SSI	637/28 724 (2.2)	637/28 724 (2.2)			
Studies that use a formal definition of SSI (*n* = 8)					
SSI	2497/34 332 (7.3)	2464/34 171 (7.2)	33/161 (20.5)	26/161 (16.1)	7/161 (4.3)
Superficial SSI	1060/25 753 (4.1)	1060/25 753 (4.1)			
Deep SSI	614/25 753 (2.4)	614/25 753 (2.4)			
Studies that used an author definition of SSI (*n* = 4)					
SSI	184/3523 (5.2)	184/3523 (5.2)			
Superficial SSI	27/2939 (9.2)	27/46 (59)			
Deep SSI	19/2939 (6.5)	19/46 (41)			

**FIGURE 3:**
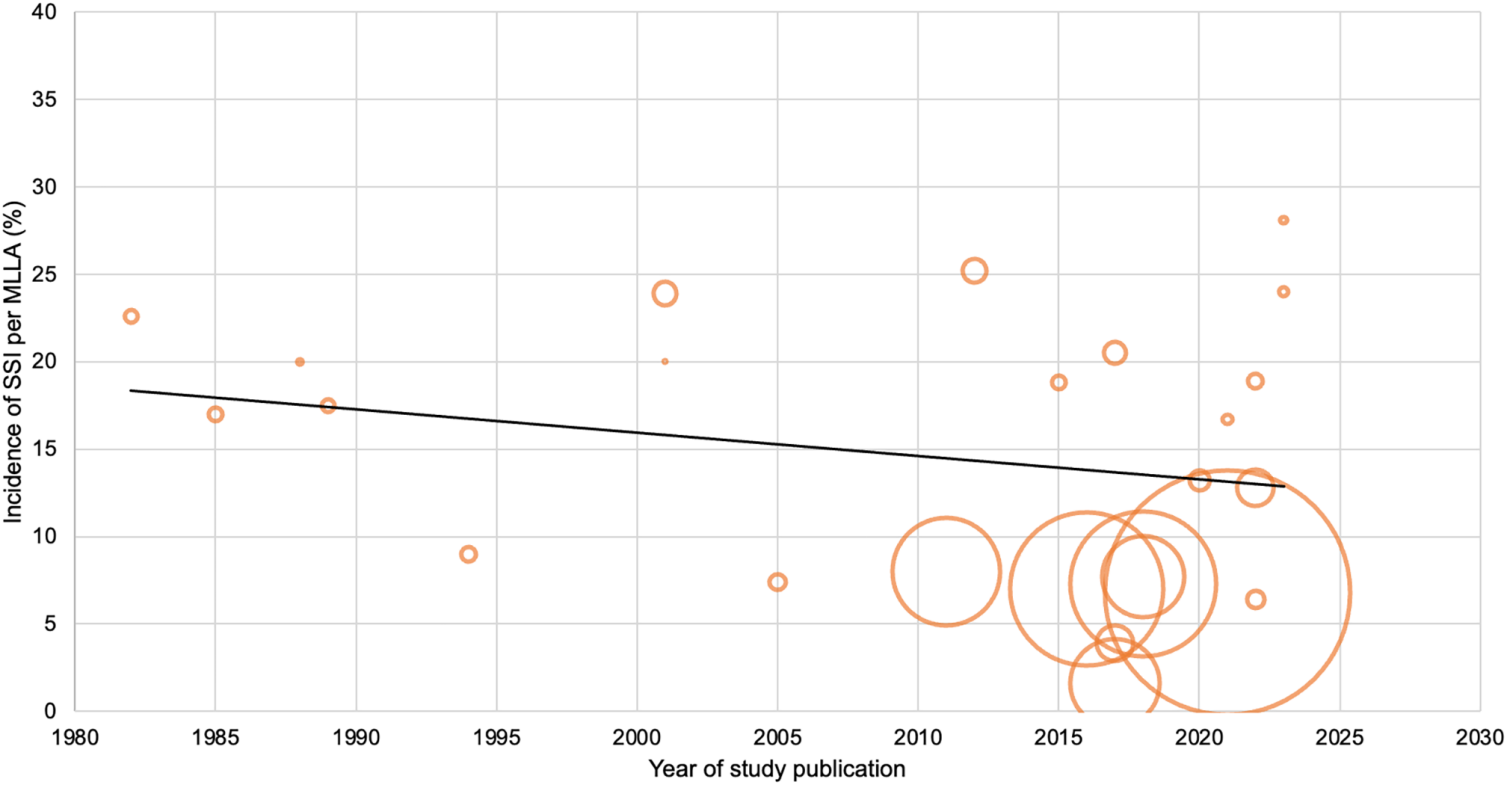
Bubble plot of the incidence of SSI (%) by year of study publication. The line of best fit shows the incidence of SSI per MLLA reduces with year of publication, and the area of the circle is proportionate to the number of patients in each study.

The year of publication for each study was used as a surrogate for the date of surgery. The incidence of SSI per MLLA decreased over time. Figure 3 shows the incidence of SSI per MLLA by year of study publication.

### 3.4 Incidence of SSI within 30 days

Ten observational studies and 2 RCTs reported the incidence of SSI within 30 days. Overall, there were 2799 SSIs reported in 40 038 MLLAs within 30 days. This equates to an SSI incidence of 7.0%. The incidence of superficial and deep SSIs within 30 days was 3.8% (1092/28724) and 2.2% (637/28724), respectively.

### 3.5 Randomised control trials

In RCTs, the incidence of SSIs during the study follow‐up period was 18.8% (50/266). The incidence of superficial and/or deep SSIs was not reported by any of the RCTs. The control arms of the RCTs experienced a higher incidence of SSIs (16.2%; 43/266) than the intervention groups (2.6%; 7/266).

SSI was a primary outcome in all the RCTs. Of the four RCTs, the underlying pathology was diabetes in one study, PAD only in two of the studies and both in one study. The incidence of SSI was similar in the RCTs which included diabetes as the underlying pathology (24/136, 17.6%) compared with the studies that only included patients with PAD (26/130, 20%).

Skin preparation was evaluated in two of the studies (preparation vs. no preparation, and chlorhexidine vs. povidone‐iodine), antibiotic therapy in one study (24 h vs. 5‐day antibiotic prophylaxis), closed incision negative wound therapy (CiNPWT) versus standard dressing in one study and one‐ versus two‐staged amputations for patients with diabetes‐related amputations in one study. Two of the RCTs used a formal definition to report SSI (ASEPSIS score and the CDC criteria).

### 3.6 Observational studies

In observational studies, the overall incidence of SSIs was 7.1% (3578/50104). In studies that included patients with both PAD and diabetes as the underlying pathology, the incidence of SSIs was 6.8% (1386/20243) in patients with diabetes, compared with 8.7% (733/8412) in studies where the underlying pathology was reported as PAD. Studies which used an existing SSI definition (*n* = 7) reported an SSI rate of 7.3% (2464/34171) and those which used an author definition reported an SSI rate of 5.2% (184/3523). Studies that had SSI as a primary outcome had a similar overall reported SSI incidence when compared with studies where SSI was not a primary outcome (7.1% and 8.5%, respectively). Six studies reported the severity of SSI, the incidence of superficial and deep SSI in these studies was 3.8% and 2.2%, respectively.

#### 3.6.1 Use of antibiotic prophylaxis

The use of prophylactic antibiotics was sporadic and inconsistent when reported. Sixteen studies did not record whether antibiotics were used or not. Four reported the use of pre‐operative antibiotics (before knife to skin) alone, 3 studies used pre‐ and post‐operative antibiotics using various preparations and durations (1–15 days), one study reported that antibiotic use was surgeon dependent but was not recorded and another 44/63 patients received some form of antibiotic therapy, but the type and duration was not recorded. One of these studies investigated the impact of extended post‐operative antibiotic prophylaxis and found significant SSI reduction in those that received them for 5 days.^31^

## 4 DISCUSSION

This systematic review has identified 25 studies with a combined SSI incidence of 7.2% and a 30‐day SSI incidence of 7.0%, following MLLA performed related to diabetes or underlying PAD. This highlights the significance of this complication in this cohort of patients. The studies included in this review were published over 40 years.

Observational studies reported a lower overall incidence of SSI following MLLA when compared with RCTs. However, from the studies, only four were RCTs. These RCTs used protocol‐driven follow‐up methods, while the observational studies often relied on hospital or national database records which may inconsistently report patient outcomes.^39^ Of the observational studies included in this review, five used data from national registries.^15^, ^21^, ^23^, ^24^, ^35^ Registries are often subject to inaccuracies and missing data which could explain the lower reported incidence of SSI.^40^ Furthermore, those with less severe SSI usually receive treatment in the primary care setting, and this information may also be missed in retrospective observational studies due to the nature of follow‐up.^41^ A similar trend has been reported in a systematic review of groin wound SSI following vascular surgery.^42^

The incidence of SSI according to severity(superficial or deep) was reported in six of the studies.^15^, ^16^, ^19^, ^21^, ^23^, ^33^ In these studies, there was a higher reported incidence of superficial SSIs. Deep SSIs are more likely to require intervention compared a superficial SSI.^43^ This could have a significant impact on a patient's quality of life, psychology, rehabilitation potential and ability to use a prosthesis.^34^, ^44^ SSIs in patients following vascular surgery have also been identified to substantially increase the costs for healthcare systems.^45^

Similar incidences of SSI were reported in the studies grouped according to underlying disease (PAD and/or diabetes). Two studies assessed MLLAs in diabetic patients, yet there were differences in patient cohorts.^15^, ^20^ One study looked at all diabetic patients undergoing MLLA,^15^ whilst the other concentrated on diabetic patients with wet gangrene,^20^ which is likely to carry a different baseline risk for developing an SSI. Furthermore, the degree and pattern of ischaemia were not reported uniformly for all the patients, and this may have an impact on post‐operative wound healing. Previous studies have highlighted that perfusion of the stump likely contributes to increased rates of stump breakdown and infection in all patients, often necessitating surgical revision,^7^, ^46^ and diabetes is known to be a risk factor for SSI. The precise contribution of both factors in this patient cohort warrants further investigation. A study with robust follow‐up is essential to accurately determine the discrepancy in SSI rates following MLLA among patients undergoing amputation due to PAD, diabetes‐related complications or both.^47^

The lower incidence of SSIs in patients with above‐knee amputations (compared with BKA/TKA) has been reported consistently in previous studies and an NCEPOD report.^8^, ^48^, ^49^ The decision to have a more distal amputation is multifactorial. Surgeon and patient preference for a longer stump which is more functional may impact the decision to undergo a TKA or BKA compared with an AKA.^50^ Also, for individual vascular units in the UK, a BKA to AKA ratio of greater than 1 is a quality performance indicator.^51^

The control arms of the RCTs consistently reported higher incidence of SSI than the intervention arms. The RCTs each evaluated a different intervention, from CiNWPT, antibiotic prophylaxis and different skin preparations pre‐operatively.^20^, ^29^, ^31^, ^34^ All the RCTs reported SSIs as a primary outcome and were published from 1989 to 2023. Reviewers noted significant concerns when evaluating the risk of bias in two of the RCTs. One trial exhibited a high risk of bias due to deviations from the intended intervention^29^ while another faced issues related to the selection of reported results.^20^ Despite concerns regarding some of the methodology in these studies, the incidence of SSI across all four RCTs remained similar. The scarcity of recent RCTs in this field highlights further studies are required to determine the best interventions to prevent SSI post‐MLLA in this patient group. Vascular surgeons have demonstrated a willingness to randomise patients to interventions and reduce SSI.^52^

There was heterogeneity in the criteria used to identify SSIs across the studies. Notably, studies which used the CDC criteria and ASEPSIS score reported a higher incidence of SSI in comparison with studies that used an author‐specified definition. Differences in SSI reporting due to the adoption of different definitions have been previously acknowledged.^42^ Also, it is important to note that several studies used data from the ACS‐NSQIP database and whilst this is validated and utilises the CDC criteria, it is not without its limitations.^15^

Included studies were published over 35 years, during which significant changes occurred in surgical practices for both the prevention and management of SSIs. There was also heterogeneity in the criteria used to diagnose and report SSIs across the included studies. This variation in definitions and methodologies made it difficult to directly compare SSI incidence, affecting the overall reliability of our conclusions. Nearly half of the studies included were found to possess a high risk of bias and low quality. However, their inclusion was thought necessary due to the scarcity of literature on this topic and the incidence of SSI was similar in the RCTs, despite the methodological concerns. The factors which influence the incidence of SSIs were not reported in most of the reviewed studies. The lack of uniformity in presenting data on patient‐specific factors, such as smoking or diabetic status, limited our ability to explore associations between these variables and the incidence of SSI. Our understanding of the intricate relationships between these factors and SSI incidence therefore remains limited and supports the adoption of a standardised reporting system.

## 5 CONCLUSIONS

The combined incidence of SSI following MLLA undertaken secondary to underlying PAD or diabetes reported extracted from the literature was 7.2%. The incidence of SSI in patients undergoing TKA or BKA is higher than those undergoing AKA. There is heterogeneity in the reporting of SSIs in this patient cohort in the existing literature. The incidence of SSI in observational studies is lower than those reported in RCTs. Higher quality evidence is required to ascertain the true incidence of SSI in this population.

## AUTHOR CONTRIBUTIONS

**Nina Al‐Saadi:** Conceptualization; data curation; formal analysis; investigation; methodology; project administration; writing – original draft; writing – review and editing. **Khalid Al‐Hashimi:** Data curation; formal analysis; methodology; writing – original draft. **Matthew Popplewell:** Conceptualization; supervision; validation; writing – review and editing. **Ismay Fabre:** Conceptualization; project administration; writing – original draft. **Brenig Llwyd Gwilym:** Conceptualization; project administration; writing – original draft; writing – review and editing. **Louise Hitchman:** Conceptualization; project administration; writing – original draft; writing – review and editing. **Ian Chetter:** Conceptualization; supervision; validation; writing – review and editing. **David C. Bosanquet:** Conceptualization; supervision; validation; writing – review and editing. **Michael L. Wall:** Conceptualization; supervision; validation; writing – review and editing.

## CONFLICT OF INTEREST STATEMENT

The authors declare no conflicts of interest.

## Data Availability

The data that support the findings of this study are available from the corresponding author upon reasonable request.
